# 

**Published:** 1902-08-09

**Authors:** 


### NOTICE TO CORRESPONDENTS.

All MSS., Letters, Books for Review, and other matters intended for the Editor should be addressed The Editor, "The Hospital," The Hospital Building, 28 & 29 Southampton Street, Strand, W.C.
The Editor cannot undertake to return rejected MS., even when accompanied by stamped directed envelope.

### Notice to Advertisers and Subscribers.

All Advertisements, Orders for Copies of The Hospital, and Business Communications should be addressed to **THE MANAGER, (Not to the Editor)**, The Hospital, 28 & 29 Southampton Street, Strand, London, W.C.
The Cost of Subscription, post free, is as follows.—Per week, 2½d.; per quarter, 2s. 8d.; per half-year, 5s. 3d.; per annum, 10s. 6d. Foreign:—Per annum, 15s. 2d., payable, in all cases, in advance.

**NOTICE.—Vol. XXXI. of "The Hospital" (October 1901, to March 1902), in handsome cloth binding is now on sale at the office of this Journal, price, 7s. 6d. Cases for binding the Numbers contained in this Volume 1s. 6d. Index to Vol. XXXI., 2½d., post free.**

**The Monthly Part for August is now ready. Price 6d., by post 8d.**

### SCRAPS AND GLEANINGS.

The designs for a new hospital on the present site of the Manchester Royal Infirmary, which have been prepared by Messrs. Simpson and Allen, and, having been approved by the Board of Management, are now being submitted to the trustees, provide for 452 beds, as compared with 292 beds now available. The architects have adopted the pavilion plan, and estimate that the cost of the erection of the new buildings will be £200,000. This sum includes the expense of providing temporary accommodation during the process of reconstruction, but is exclusive of the cost of furniture and fittings; the architects state that the time occupied by the reconstruction will be about six years, as it is intended to rebuild in sections so as to interfere as little as possible with the work of reconstruction.

### THE STUDENT OF MEDICINE.

### APPOINTMENTS.

**E. P. Baumann**, M.D.Edin., M.R.C.S., appointed Medical Registrar to the Hospital for Sick Children, Great Ormond Street. **John F. H. Broadbent**, M.D., M.R.C.P., appointed Assistant Physician to the London Fever Hospital. **Wm. R. Gibson**, M.B., Ch.B.Glasg., appointed Medical Officer of Health to the Cheadle Rural District Council. **John Harley Gough**, F.R.C.S. Edin., L.R.C.P.Lond., appointed Physician to the Torbay Hospital, Torquay. **Arthur Greene**, M.D.Dub., appointed House Surgeon, Royal South London Ophthalmic Hospital, Southwark, S.E. **Sydney Herbert Long**, M.B., B.C.Cantab., appointed Medical Officer of Health to the St. Faith's Rural District Council. **J. P. Maynard**, M.B., F.R.C.S., Major I.M.S., appointed Ophthalmic Surgeon and Professor of Ophthalmic Surgery in the Medical College, Calcutta. **A. E. Sansom**, M.D., appointed Consulting Physician to the London Hospital. **J. W. S. Seccombe**, M.R.C.S., L.R.C.P., appointed House Physician to St. George's Hospital. **J. F. Skrimshire**, B.C.Camb., appointed District Medical Officer of the Walsingham Union. **W. A. Valentine**, M.D.Univ. Dub., Trin. Coll., appointed District Medical Officer and Public Vaccinator of the Northam District of the Bideford Union. **J. Wallace**, M.D.Aberd., appointed Certifying Factory Surgeon for the Stonehaven District of Kincardineshire. **C. Woodhouse**, M.D., B.C.Cantab., appointed Certifying Factory Surgeon for the Urban Districts of Esher and the Dittons, East and West Molesey, and Walton-on-Thames.

**"The Hospital" Institutional Library.**

"Hospitals and Asylums of the World." 4 Vols., with a Portfolio of Plans, complete £12 12s.

"Burdett's Hospitals and Charities, 1902." 5s.

"Cottage Hospitals, General, Fever, and Convalescent." 10s. 6d.

"Hospital Expenditure: The Commissariat." 2s. 6d.

"A Legal Handbook for the Use of Hospital Authorities." 2s. 6d.

"The Cottage Hospital Case Book or Register of Patients." 10s. and 12s. 6d.

"The Furnishing and Appliances of a Cottage Hospital." 6d.

All these are published by the Scientific Press, Ltd., and may be obtained through any bookseller, or direct from the publishers, 28 and 29 Southampton Street, Strand, London, W.C.

